# Assessing the outcomes of everolimus on renal angiomyolipoma associated with tuberous sclerosis complex in China: a two years trial

**DOI:** 10.1186/s13023-018-0781-y

**Published:** 2018-03-27

**Authors:** Yi Cai, Hao Guo, Wenda Wang, Hanzhong Li, Hao Sun, Bing Shi, Yushi Zhang

**Affiliations:** 1 Department of Urology, Peking Union Medical College Hospital, Chinese Academy of Medical Sciences and Peking Union Medical College, 1 Shuaifuyuan Road, Beijing, 100730 China; 2 Department of Radiology, Peking Union Medical College Hospital, Chinese Academy of Medical Sciences and Peking Union Medical College, Beijing, 100730 China

**Keywords:** Angiomyolipoma, Tuberous sclerosis complex, Everolimus, Treatment outcome, Safety

## Abstract

**Background:**

Tuberous sclerosis complex (TSC) is a rare autosomal dominant genetic disorder characterized by the development of numerous benign tumors. Renal angiomyolipoma (RAML) occur in up to 80% of TSC patients, which is a leading cause of TSC-related death in adult patients. The aim of the study was to evaluate the efficacy and safety profiles of everolimus in Chinese patients of TSC associated with RAML(TSC-RAML).

**Methods:**

In this 2-years, nonrandomized, open-label trial, 18 patients of TSC-RAML, with at least one RAML 3 cm or larger in its longest diameter, were enrolled to assess the efficacy and safety of everolimus therapy in Chinese patients. Everolimus was administered for the first 12 months only. The primary endpoint was a reduction of 50% or more relative in RAML volume to the baseline in the absence of new RAML ≥1 cm and no RAML-related bleeding of grade ≥ 2. The secondary endpoints included: safety, lung function and skin lesions response rate. Serial computed tomography of RAML, magnetic resonance imaging of brain lesions and pulmonary-function tests were performed. Adverse events were investigated using CTCAE v4.0. All analyses used a significance level of 0.05 and were generated in SPSS19.0 software.

**Results:**

The proportion of patients who achieved ≥50% reduction from baseline in the sum of volumes of target lesions increased from 52.94% at 3 months, to 58.82% and 66.67% at months 6 and 12, respectively. During the period of everolimus therapy, among patients with lymphangioleiomyomatosis, the mean forced expiratory volume in 1 s (FEV1) increased by 276 ± 78 ml (*P* < 0.001), the forced vital capacity (FVC) increased by 433 ± 170 ml (*P* < 0.001), and the residual volume decreased by 408 ± 243 ml (*P* = 0.009), as compared with baseline values. The angiomyolipoma volume and the lung function approached, but did not completely return to, the baseline values. The skin lesions response rate was 37.5% after 12 months of therapy falling to 21.4% at 12 months after stopping everolimus. The most common adverse events were mucositis oral, irregular menstruation, abdominal pain, hypertriglyceridemia and headache. The most common grade 3 adverse events were irregular menstruation and mucositis oral. In addition, one patient died from RAML spontaneous haemorrhage during treatment with everolimus, even with reduction in RAML volume of 60.68% at 3 months. A second death was due to epithelioid RAML progression, with metastasis to multiple retroperitoneal lymph node, who died from severe infection one month after surgery.

**Conclusions:**

Angiomyolipomas regressed somewhat during everolimus therapy but tended to increase in volume after the therapy was stopped. Everolimus was well tolerated and showed promising activity in Chinese patients with TSC-RAML, however, we should alert the life-threatening hemorrhage of large RAML in the early period and the lymph node metastasis of epithelioid RAML.

**Trial registration:**

ChiCTR-OPC-14005488. Registered November 17, 2014.

**Electronic supplementary material:**

The online version of this article (10.1186/s13023-018-0781-y) contains supplementary material, which is available to authorized users.

## Background

Tuberous sclerosis complex (TSC) is an autosomal dominant syndrome affecting 1~ 2 million people worldwide [1]. It is characterized by prominent neurodevelopmental features and by tumors that develop in the brain, skin, heart, kidneys and lungs [1]. Renal angiomyolipoma (RAML) develop in approximately 80% of adults and adolescents with TSC [1, 2]. RAML associated with tuberous sclerosis complex (TSC-RAML) are characterized as multiple and commonly bilateral lesions that consist of blood vessels, smooth muscles, and adipose tissues [2]. TSC-RAML typically grow over time, presenting of arterial hypertension and risk of potentially life-threatening hemorrhage, which is the leading cause of TSC-associated death in adult patients [3]. To date, the main therapeutic options are embolisation, elective surgery, and emergency nephrectomy in cases of uncontrollable haemorrhage in China [4].

The majority of individuals with TSC have mutations in either the TSC1 or TSC2 genes, and subsequent somatic mutation results in constitutive activation of mammalian target of rapamycin (mTOR), a critical regulator of cell growth, proliferation, and angiogenesis [5–7]. Bissler JJ and colleagues demonstrated everolimus, a mammalian target of rapamycin (mTOR) inhibitor, could significant reduction in renal angiomyolipoma volume compared with placebo in Western populations [8]. The International tuberous sclerosis complex consensus conference held in 2012 recommended mTOR inhibitors as the first-line treatment for RAML when enlarged to 3 cm or more, even when asymptomatic [9]. Transcatheter arterial embolization and partial nephrectomy are recommended as second-line treatments [9]. However, we lack data of the efficacy and safety of everolimus in treatment of TSC-RAML in Chinese patients.

The objective of the present study was to evaluate the efficacy and safety of everolimus for TSC-RAML in Chinese patients.

## Methods

### Patients

This trial was a 2-years, nonrandomized, open-label trial, phase 2 study (ChiCTR-OPC-14005488, https://www.chictr.org.) conducted at Peking Union Medical College Hospital start from December 2014. From December 2014 to November 2015, patients were included if they met the following inclusion criteria: (1). Men or women (not pregnant) ≥18 years; (2). Clinical and/or genetic diagnosis of TSC; (3). CT or MRI shows one or more TSC-RAMLs with the longest diameter ≥ 3 cm; (4). Without RAML bleeding or embolism in the past 6 months. Patients were excluded from the study if they met the following exclusion criteria: (1). Age < 18 years; (2). Women who plan to be pregnant or have been pregnant or lactating; (3). CT or MRI shows the longest diameter of RAML < 3 cm; (4). Patients expected to undergo surgery or embolization therapy during the trial; (5). History of coronary heart disease, myocardial infarction or cerebral infarction related to atherosclerosis; (6). History of RAML bleeding or embolism in the past 6 months; (7). Impaired lung function defined as following: For patients without lymphangioleiomyomatosis (LAM): Known impaired lung function (e.g. FEV1 or DLco ≤70% of predicted); For patients with LAM: DLco ≤35%, or O_2_ saturations below normal at rest, or O_2_ saturation ≤ 88% on 6 min walking test with up to 6 l O_2_/min nasal oxygen; (8). Severe hematological disease or liver function abnormality (such as aminotransferase > 2.5 times of normal upper limit, serum bilirubin > 1.5 times of normal upper limit, hemoglobin < 9 g/dL, platelet < 80,000/mm^3^, or absolute neutrophil count < 1000/mm^3^); (9). Concomitant severe infection before or during the trial; (10). Previous organ transplantation; (11). History of other surgeries (involving entry into body cavity or suture) in the past 2 months; (12). Previous mTOR inhibitor treatment (such as sirolimus and everolimus); (13). Use of investigational drug within 30 days; (14). Poor control of hyperlipidemia: fasting serum cholesterol > 300 mg/dL (or > 7.75 mmol/L), fasting triglyceride > 2.5 times of normal upper limit; (15). Poor control of diabetes: fasting blood glucose > 1.5 times of normal upper limit; (16). Patients with bleeding tendency or using oral anti-vitamin K drugs (except low-dose warfarin); (17). History of HIV seropositivity; (18). Active hepatitis; (19). Patients who cannot participate in regular visits and follow-up; (20). Patients who are not suitable for MRI examination (such as excessive obesity, mental disorders, bullet fragment in body, stent and pacemaker); (21). Serum creatinine > 1.5 times of normal upper limit. This study protocol was approved by the Human Ethics Committee of Peking Union Medical College Hospital, before the first patient was enrolled.

### Study design

This study includes two periods: the core period and the extended period. Everolimus was administered for the first 12 months only. The core period of the study lasts for 1 year, and then all possible patients will continue the next 1-year extended observation. The dose is initiated with 10 mg daily by oral, and then titrated to the target tough blood concentration of 5–15 ng/ml during the first 3 months and maintained throughout the observation, with dose modifications allowed on the basis of safety findings. Concomitant use of strong inhibitors or inducers of cytochrome P450 3A4 or p-glycoprotein (PgP) was to be avoided during the study; use of antiproliferative agents other than study drug was prohibited. All planed visits and assessments are listed in Additional file 1: Table S1. The primary efficacy endpoint was the proportion of patients with a confirmed angiomyolipoma response, defined as a reduction in angiomyolipoma volume (sum of volumes of all target angiomyolipomas > 1 cm identified at baseline) of 50% or more relative to baseline and absence of angiomyolipoma progression. In addition, RAML response requires satisfying the following criteria: (1) No new RAML ≥1.0 cm in longest diameter are identified; (2) The patient does not have any RAML-related bleeding of grade ≥ 2 as defined by the National Cancer Institute Common Terminology Criteria for Adverse Events, version 4.0 (NCI-CTCAE v.4). Key secondary endpoints were time to angiomyolipoma progression and skin lesions response rate. Computed Tomography (CT) or magnetic resonance imaging(MRI), same modality used throughout the study for each patient, used to calculate RAMLs volume at baseline and repeated at 3, 6, 12, 18 and 24 months after the start of treatment. Skin lesions resulting from tuberous sclerosis complex include hypomelanotic macules, the shagreen patch, periungual or subungual fibromas, and facial angiofibromas, forehead plaques, or both, and were assessed at baseline and repeated at 3, 6, 12, 18 and 24 months after the start of treatment using the seven-point grading scale Physician’s Global Assessment of Clinical Condition [10, 11] (Additional file 2: Table S2). Patients with lymphangioleiomyomatosis underwent pulmonary-function testing at baseline, 12 months, and 24 months. Adverse events were monitored throughout the study and graded according to the Common Terminology Criteria for Adverse Events v4.0 via patient-reported or caregiver-reported responses as well as investigator assessment.

### Statistical analysis

Data are expressed as the mean ± standard deviation (M ± SD) or n (%). Statistical significance was determined by paired or unpaired Student’s t-test in cases of standardized expression data. The SPSS software package (version 17.0) was used for all statistical analysis. *P* value< 0.05 was defined as statistically difference.

## Results

### Characteristics of the patients

Eighteen patients were enrolled in this trial. Their demographic details and disease characteristics are summarized in Table 1. The median age was 29 years, 9 patients were under 30 years of age. All 18 patients underwent genotyping by next-generation sequencing and confirmed all for TSC2 mutation (Additional file 3: Table S3). Six female patients were diagnosed for lymphangioleiomyomatosis (LAM) while one patient presence of subependymal giant cell astrocytoma. Two patients had undergone embolisation, and two patients had previously undergone partial nephrectomy while another two undergone unilateral nephrectomy. In addition, there were no large, at least 5 mm or larger in size, intra-renal aneurysm in all 18 patients. Three patients left the study during the first year: one had a unilateral renal hemorrhage and died at fourth fellow-up month, one died due to epithelioid RAML progression at eleven fellow-up month, and one did not comply with the protocol. Fifteen patients underwent the 12-month evaluation. One withdrew from the study after the 18-month visit to continue everolimus therapy off-label because of worry about tumor progress fast, leaving 14 patients at the 24-month assessment of angiomyolipoma.

**Table 1:** Baseline patient demographic and disease characteristics

	Everlimus (*N* = 18)
Age in years, median (range)	29(20~ 46)
< 30 year	9(50%)
≥ 30 year	9(50%)
Sex	
Women	12(66.7%)
Men	6(33.3%)
Gene mutation	
TSC1	0(0%)
TSC2	18(100%)
Diagnosis of lymphangioleiomyomatosis	6(33.3%)
Skin lesion(≥1)	18(100%)
Presence of subepedymal giant cell astrocytoma	1(5.6%)
Longest diameter of the largest renal angiomyolipoma lesion	
≥ 8 cm	14(77.8%)
≥ 4 cm and < 8 cm	4(22.2%)
< 4 cm	0(0%)
Sum of volumes of target renal angiomyolipoma lesions, cm^3^	
Mean (SD)	1974 (2406)
Median (range)	1247 (60.77~ 9079)
Bilateral angiomyolipoma	16(88.9%)
Number of target angiomyolipoma lesions	
1~ 2	11(61.1%)
3~ 4	7(38.9%)
Previous angiomyolipoma therapy	
Surgery/invasive procedure	6(33.3%)
Renal embolisation	2(11.1%)
Partial nephrectomy	2(11.1%)
Nephrectomy	2(11.1%)
Medication	0(0%)

### Treatment efficacy

**Fig. 1:**
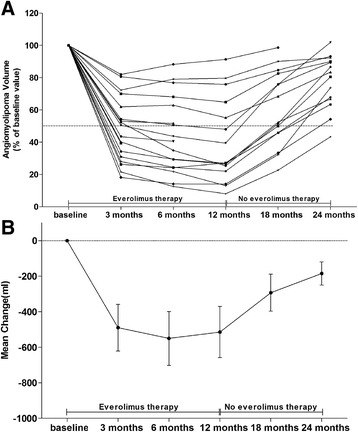
Renal Angiomyolipoma Volume in the Patients with the Tuberous Sclerosis Complex during the Study. Panel **a** shows the renal angiomyolipoma volume at each visit is expressed as a percentage of the baseline size. The dashed line represents 50% of the baseline value; data below the line indicate that the mean angiomyolipoma volume was reduced by 50% or more. Panel **b** shows the mean change (in milliliters) from the baseline values for renal angiomyolipoma volume. I bars indicate the standard errors

**Fig. 2:**
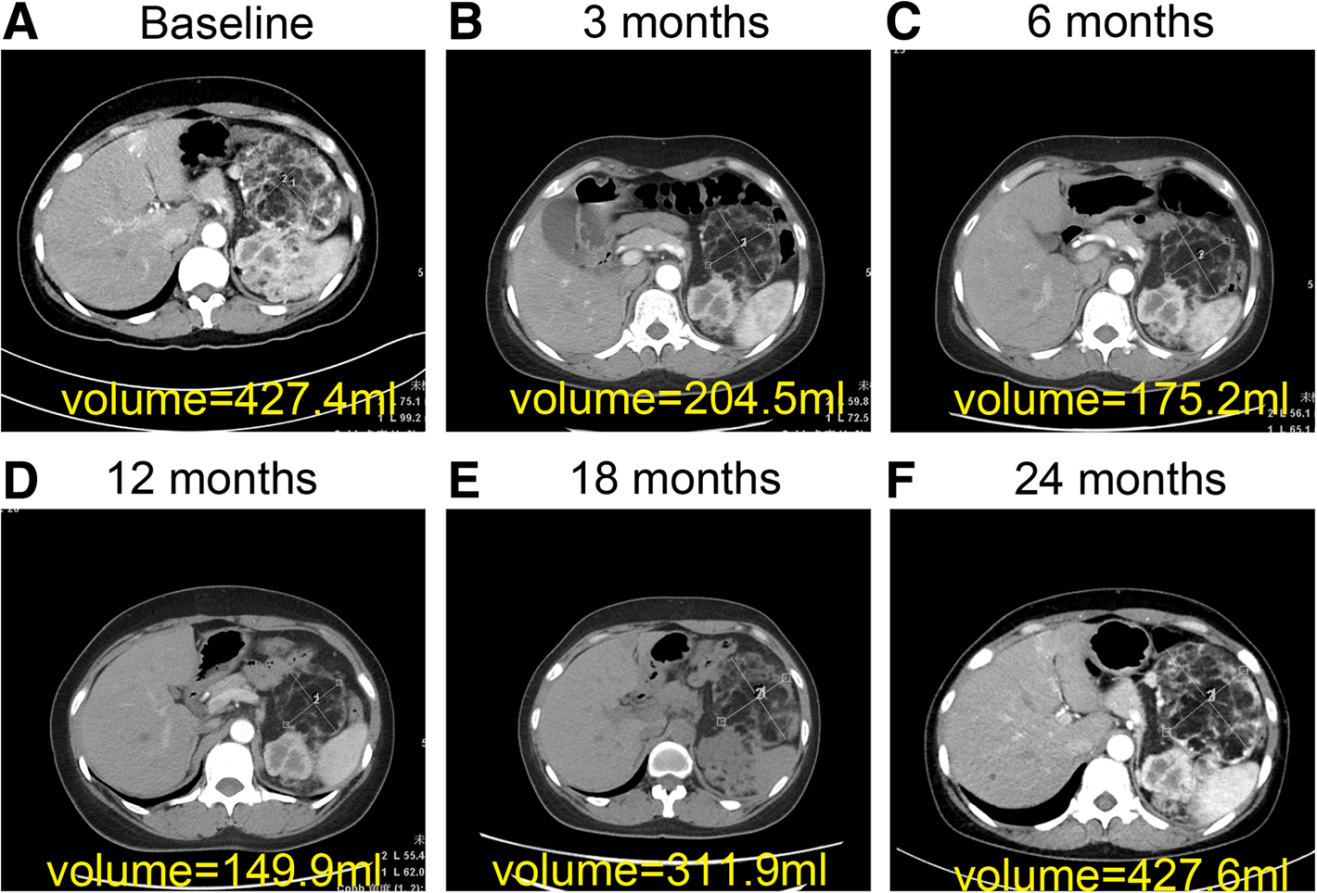
Target Renal Angiomyolipomas of a Patient with the Tuberous Sclerosis Complex. Panel **a** shows the target renal angiomyolipoma volume (in milliliters) at baseline. Panel **b**, **c** and **d** shows the target renal angiomyolipoma volume (in milliliters) after 3, 6 and 12 months of everolimus therapy. Panel **e** and **f** shows the target renal angiomyolipoma volume (in milliliters) at 6 and 12 months after stopping everolimus

The proportion of patients who achieved ≥50% reduction from baseline in the sum of volumes of target lesions increased from 52.94% (9/17) at 3 months, to 58.82% (10/17) and 66.67% (10/15) at months 6 and 12, respectively. Primary-tumor shrinkage was most rapid during the initial 3 months of treatment, with evidence of a sustained response at subsequent time points during the core treatment phase. Median time to angiomyolipoma response for everolimus was 3.0 months. The mean renal angiomyolipoma volume at baseline was 1974 ± 2406 ml (Table 1). After 12 months of therapy, the mean volume decreased to 41.14 ± 26.54% of the baseline volume (*P*<0.002) (Table 2 and Fig. 1). At 6 and 12 months after stopping everolimus, the mean angiomyolipoma volume had increased to 60.67 ± 23.28% of the baseline volume (*P* = 0.006) and 77.62 ± 16.66% of the baseline volume (*P* = 0.014), respectively (Table 2 and Fig. 1). The response of a renal angiomyolipoma along with time of everolimus therapy, visualized on CT, is shown in Fig. 2. The current trial shows that everolimus are effective in reducing angiomyolipoma size in tuberous sclerosis in Chinese patients.

**Table 2:** Response of angiomyolipoma volume to everolimus therapy

	3 months	6 months	12 months	18 months	24 months
Patients (n)	18	17	15	15	14
No. of response (n,%)	9 (52.94%)	10 (58.82%)	10 (66.67%)	5(33.33%)	1(7.14%)
^a^% of baseline value (Mean ± SD,%)	47.73 ± 19.81	44.27 ± 23.69	41.14 ± 26.54	60.67 ± 23.28	77.62 ± 16.66
*P* value for change from baseline value	0.002	0.002	<0.001	0.006	0.014

^a^the average percentage change of baseline in the total volume of all target AML lesions

**Fig. 3:**
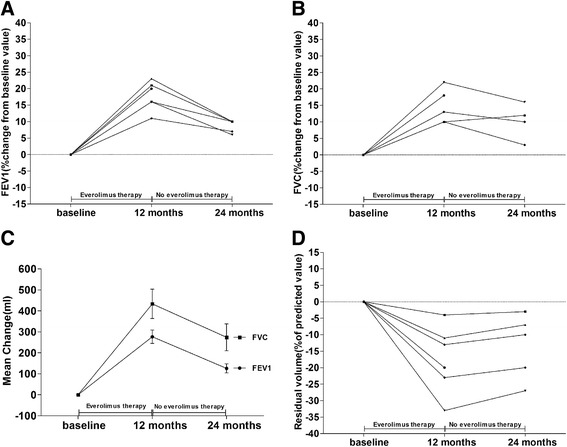
Pulmonary Function in Patients with Lymphangioleiomyomatosis. Panel **a** shows the forced expiratory volume in 1 s (FEV1) for each patient. Panel **b** shows the forced vital capacity (FVC) for each patient. Panel **c** shows the mean change (in milliliters) from the baseline values for FEV1 and for FVC. I bars indicate the standard errors. Panel **d** shows the residual volume for each patient

Pulmonary functional data for 6 female patients with lymphangioleiomyomatosis are listed in Table 3. All six patients had never been smokers. At enrollment, spirometric measurements were normal in one patients, revealed moderate airflow obstruction (forced expiratory volume in 1 s [FEV1], 50 to 70% of the predicted value) in three patients, and indicated severe airflow obstruction (FEV1 < 50% of the predicted value) in two patients. During everolimus therapy, the mean FEV1 increased from the baseline mean by 276 ± 78 ml at 12 months (*P* < 0.001), while the mean FVC increased from the baseline mean by 433 ± 170 ml at 12 months (*P* < 0.001) (Fig. 3a and c). After 1 year of everolimus therapy, the FEV1 and FVC in these patients were significantly improved (Table 3). Twelve months after stopping everolimus, the mean FEV1 was 126 ± 48 ml greater than the mean baseline value (*P* = 0.004), while the mean FVC was 274 ± 142 ml greater than the mean baseline value (*P* = 0.008) (Fig. 3b and c). The mean percent of the predicted FEV1 value were significantly improved at 12 months (*P* < 0.001) and at 24 months (*P* = 0.008) (Table 3). The mean percent of the predicted FVC value were significantly improved at 12 months (*P* < 0.001) and at 24 months (*P* < 0.001) (Table 3). The mean residual volume fell by 408 ± 243 ml after 1 year of everolimus therapy, as compared with the baseline value (*P* = 0.009) (Table 3). The mean percent of the predicted residual volume were significantly improved at 12 months (*P* < 0.001) and at 24 months (*P* < 0.001) (Table 3). Neither the DLco nor total lung capacity changed significantly during the study (Table 3).

**Table 3:** Pulmonary Structural and Functional Characteristics of Patients with Lymphangioleiomyomatosis

Value	Baseline (*n* = 6)	12 months (*n* = 6)	24 months (*n* = 5)	Change from Baseline	
				12 months (*n* = 6)	24 months (*n* = 5)
FEV1					
Least-square mean — liters	1.59 ± 0.41	1.87 ± 0.47	1.65 ± 0.45	0.28 ± 0.08 (*P*<0.001)	0.13 ± 0.05 (*P* = 0.004)
Percent of predicted value	58.51 ± 11.09	65.32 ± 12.32	59.89 ± 11.58	6.81 ± 2.28 (*P*<0.001)	3.46 ± 1.57 (*P* = 0.008)
FVC					
Least-square mean — liters	2.77 ± 0.56	3.20 ± 0.68	2.95 ± 0.66	0.43 ± 0.17 (*P* = 0.002)	0.27 ± 0.14 (*P* = 0.013)
Percent of predicted value	79.90 ± 12.68	87.16 ± 12.63	84.36 ± 13.71	7.26 ± 2.16 (*P*<0.001)	6.39 ± 1.36 (*P*<0.001)
Total lung capacity					
Least-square mean — liters	5.16 ± 0.51	5.18 ± 0.44	5.14 ± 0.49	0.03 ± 0.12 (*P* = 0.62)	0.04 ± 0.10 (*P* = 0.43)
Percent of predicted value	99.21 ± 7.01	99.70 ± 7.28	97.75 ± 7.24	0.49 ± 1.47 (*P* = 0.45)	−0.22 ± 0.78 (*P* = 0.57)
Residual volume					
Least-square mean — liters	2.39 ± 0.10	1.98 ± 0.31	2.11 ± 0.29	−0.41 ± 0.24 (*P* = 0.009)	−0.31 ± 0.23 (*P* = 0.036)
Percent of predicted value	124.56 ± 10.04	104.23 ± 8.85	112.30 ± 5.41	−20.32 ± 1.62 (*P*<0.001)	−15.79 ± 2.38 (*P*<0.001)
DLco					
Least-square mean — ml/mmHg/min	12.93 ± 2.42	13.00 ± 2.41	12.59 ± 2.39	0.07 ± 0.46 (*P* = 0.71)	−0.21 ± 0.55 (*P* = 0.43)
Percent of predicted value	53.40 ± 9.29	53.60 ± 8.70	54.27 ± 9.56	0.19 ± 1.39 (*P* = 0.74)	1.68 ± 2.11 (*P* = 0.15)

Data are listed for 6 patients with lymphangioleiomyomatosis who completed the 12-month study visit. Data for the 24-month study visit were available for 5 of these 6 patients; 1 patient had withdrawn during the second year. Changes from baseline were not significant unless otherwise indicated. *DLco* denotes diffusing capacity of the lung for carbon monoxide, *FEV1* forced expiratory volume in 1 s, *FVC* forced vital capacity

All patients had cortical tubers; 15 patients had subependymal nodules and one had subependymal giant-cell astrocytomas. There were no changes in the size of the subependymal nodules. We did not get the evaluable data of the patient with subependymal giant-cell astrocytomas as she died for RAML spontaneous haemorrhage in four months. Although this was not a study endpoint, a slight improvement in the frequency of seizures was reported by some parents.

**Fig. 4:**
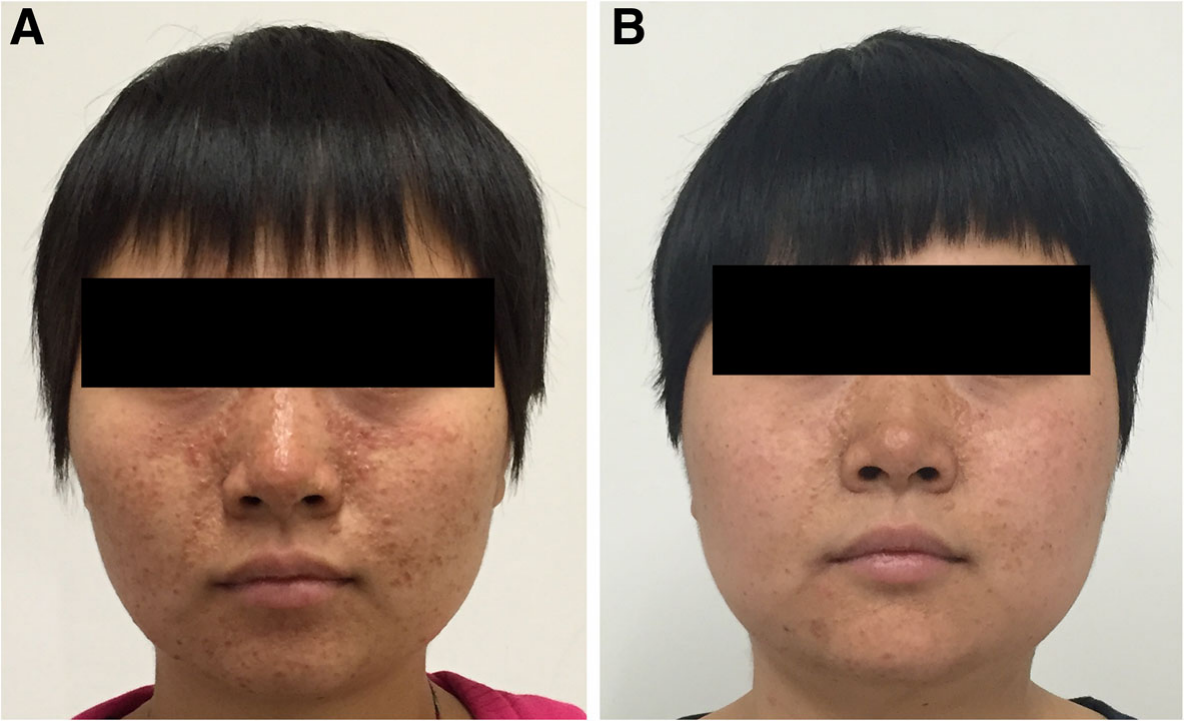
Facial angiofibromas of a Patient with the Tuberous Sclerosis Complex. Panel **a** shows the facial angiofibromas at baseline. Panel **b** shows the facial angiofibromas improvement after 12 months of everolimus therapy

Skin lesions associated with tuberous sclerosis were present at baseline in all 18 patients. Facial angiofibromas decreased in size and became paler and less rough after 12 months of therapy (Fig. 4). The skin lesions response rate was 37.5% (6 of 16) after 12 months of therapy falling to 21.4% (3 of 14) at 12 months after stopping everolimus.

### Adverse events

Adverse events were consistent with the known everolimus safety profile. The most common adverse events were mucositis oral (100%), irregular menstruation (91.7%), abdominal pain (77.8%), hypertriglyceridemia (72.2%) and headache (66.7%) (Table 4). The most common grade 3 adverse events were irregular menstruation (25%) and mucositis oral (11.1%) (Table 4). Three patients with irregular menstruation was classified as grade 3 adverse events due to persistent amenorrhea for more than 6 months. In the trial, one patient died from RAML spontaneous haemorrhage during treatment with everolimus, even with reduction in RAML volume of 60.68% at 3 months. A second death was due to epithelioid RAML progression, who underwent left radical nephrectomy and immunohistochemical examination revealed features of epithelioid AML accompany with retroperitoneal multiple lymph node metastasis. The patient died from severe infection one month after surgery.

**Table 4:** Adverse events by preferred term, regardless of relationship to everolimus

n(%)		Everolimus (*N* = 18)		
	All grades	Grade 3	Grade 4	Grade 5
Mucositis oral	18(100%)	2(11.1%)	–	–
Irregular menstruation	11(91.7%)	3(25.0%)	–	–
Abdominal pain	14(77.8%)	1(5.6%)	–	–
Hypertriglyceridemia	13(72.2%)	–	–	–
Headache	12(66.7%)	–	–	–
Diarrhea	11(61.1%)	–	–	–
Upper respiratory infection	10(55.6%)	–	–	–
Proteinuria	9(50%)	1(5.6%)	–	–
Malaise	9(50%)	–	–	–
Rash acneiform	8(44.4%)	–	–	–
Cholesterol high	8(44.4%)	–	–	–
Fever	6(33.3%)	–	–	–
Urinary tract infection	5(27.8%)	–	–	–
Hematuria	5(27.8%)	–	–	–
Alkaline phosphatase increased	5(27.8%)	–	–	–
Constipation	4(22.2%)	–	–	–
GGT increased	4(22.2%)	–	–	–
Hypophosphatemia	4(22.2%)	–	–	–
Seizures	3(16.7%)	–	–	–
Pneumonitis	2(11.1%)	1(5.6%)	–	1(5.6%)
Vomiting	2(11.1%)	1(5.6%)	–	–
Lymphocyte count decreased	2(11.1%)	–	–	–
Anemia	2(11.1%)	–	–	–
Renal hemorrhage	1(5.6%)	–	–	1(5.6%)
Neutrophil count decreased	1(5.6%)	–	–	–
Hyperuricemia	1(5.6%)	–	–	–
Creatinine increased	1(5.6%)	–	–	–

## Discussion

In the current trial, we reported the efficacy and safety of everolimus in treatment of TSC-RAML in Chinese patients for the first time. Everolimus therapy in patients with the tuberous sclerosis complex was associated with a reduction in angiomyolipoma volume, improvements in skin lesions and pulmonary function. The renal and pulmonary benefits of treatment with everolimus tended to reverse after the drug was withdrawn, though the improvements were persistent in part patients.

In patients with the tuberous sclerosis complex, renal disease is a leading cause of death or disability in adult patients [3]. TSC is a rare autosomal dominant genetic disease caused by mutations in theTSC1 gene coding for hamartin and TSC2 gene coding tuberin [5, 6]. Finding that tuberin plays an important role in mTOR signaling pathway and further identification of tuberin hamartin complex as a main inhibitor of this pathway opened up new possibilities in disease-modifying therapy for TSC patients [12, 13]. Randomized clinical trials support the use of everolimus, an inhibitor of the mammalian target of rapamycin, in the treatment of subependymal giant cell astrocytomas (SEGA), RAML and seizure related to TSC in western populations [8, 14, 15]. However, its efficacy and safety in treatment of Chinese TSC patients is unknown. In the trial, tumor partial response by RECIST criteria was observed in 52.94%, 58.82% and 66.67% at months 3, 6 and 12 in Chinese adult patients, respectively. Bissler and colleagues investigated everolimus treatment for RAML in patients with tuberous sclerosis and found 44.2%, 55% and 64.5% patients achieved ≥50% reduction from baseline in the sum of volumes of target lesions at weeks 12, 24 and 96, respectively [8, 16]. Another subgroup analysis from EXIST-1 trial showed similar results that the proportions of patients in the everolimus arm with 50% reduction in the sum of target RAML were 56.5%, 78.3% and 80.0% after 12, 24 and 48 weeks, respectively [17]. In addition, Bissler and colleagues investigated another mTOR inhibitor, sirolimus, treatment for RAML in patients with tuberous sclerosis or sporadic LAM and found a mean reduction in RAML volume of 47% at 12 months [18]. The observation that angiomyolipoma size correlates with the risk of hemorrhage suggests that maintains or reduces angiomyolipoma size may reduce the risk of bleeding. However, the mean angiomyolipoma volume had increased ranging from a rapid return to baseline dimensions to a sustained reduction in size, after the withdrawal of everolimus. The current trial shows that everolimus are effective in reducing RAML size in tuberous sclerosis in Chinese patients. But long-term maintenance of everolimus therapy is necessary in TSC-RAML patients as it increased after stopping therapy. Everolimus administration to patients with TSC-RAML over the extension phase of the EXIST-2 trial supports a long-term benefit over approximately 4 years [19]. Therefore, it seems reasonable to assume that TSC-RAML patients should be kept under mTOR inhibition for life.

Six patients with lymphangioleiomyomatosis were evaluated for pulmonary outcomes. After 12 months of everolimus therapy, the FEV1 increased by 276 ml, and the FVC increased by 433 ml, while the mean residual volume fell by 408 ml. In addition, the FEV1, FVC and residual volume remained the most improved 12 months after everolimus was stopped, as compared with the baseline values. However, neither the DLco nor total lung capacity changed significantly during everolimus therapy. In addition, facial angiofibromas decreased in size and became paler and less rough after 12 months of therapy, which remained the most improved 12 months after stopping everolimus. There was no change in subependymal nodules size during the study. However, everolimus appears to have activity in the central nervous system, on the basis of a slight improvement in the frequency of seizures was reported by two patients. In all, treatment with everolimus for 1 year resulted in an improvement in lung function and skin lesions in adults with the TSC-RAML.

Adverse events were common, consistent with the known toxicities of everolimus and mostly of low grade. The most frequent adverse reactions we recorded were mucositis oral (100%), which was observed at the start of treatment. The most common grade 3 adverse events were irregular menstruation, occurred in five out of twelve female patients (25.0%). The frequency of mucositis oral and irregular menstruation was higher than the EXIST-2 and subgroup analysis of EXIST-1 study [1, 3], which may be due to the different race of patients. Although three cases of amenorrhoea in our study resolved without intervention, surveillance for this potential side effect is warranted in women patients of child-bearing potential and needed further investigated. In the current trial, one patient died from RAML spontaneous haemorrhage during treatment with everolimus, even with reduction in RAML volume of 60.68% at 3 months. As far as we know, it is the first case reported RAML spontaneous haemorrhage during treated with everolimus. The sum of RAML volume burben is over 4000 ml in this patient, which may the major reason for RAML spontaneous haemorrhage. A second death was due to epithelioid RAML progression, with metastasis to multiple retroperitoneal lymph node, and died from severe infection one month after surgery. Epithelioid RAML is considered to be a potentially malignant tumor, which is composed of a prominent epithelioid component, with spindle and giant cells, and contains none or a minimal amount of adipose tissue. Epithelioid RAML can occur in patients both with and without tuberous sclerosis. However, half of published cases have history of tuberous sclerosis, some show metastatic potential [20, 21]. In addition, the mTOR pathway was recently found to be activated in epithelioid angiomyolipoma [22], and Wolff N, et al. reported that mTOR inhibitors, such as sirolimus or temsirolimus, in two cases of epithelioid angiomyolipoma and showed a good short term response [23]. The two patients died during the clinical trial, reminds us that the risks and benefits of everolimus need specific and careful evaluation in the real world.

Several limitations in our study should be noted: (1) it is a single center based on a small sample without placebo-controlled trial; (2) none patient with TSC1 gene mutation was included; (3) all patients were adult, so clinical trials concerning the safety of everolimus for child patients needed in the future.

## Conclusions

Collectively, the data suggest that everolimus therapy for 1 year not only resulted in a decrease in the size of angiomyolipomas, but also improved in lung function and skin lesions in adults Chinese patients with TSC-RAML. One year after the drug was discontinued, the angiomyolipoma size and the lung function approached, but did not completely return to, the baseline values. It seems reasonable to assume that TSC-RAML patients should be kept under mTOR inhibition for life. The most common adverse events were mucositis oral, irregular menstruation, abdominal pain, hypertriglyceridemia and headache. The two patient who died in the current trial reminds us that the risks and benefits of everolimus need specific and careful evaluation in the real world. In conclusion, this study shows that everolimus is a relatively safe and efficacious in treatment of TSC-RAML in Chinese adult patients.

## Additional files

Additional file 1: Visit Schedule and Assessments. (DOCX 73 kb)

Additional file 2: Physician’s Global Assessment of Clinical Condition. (DOCX 98 kb)

Additional file 3: Mutations detected by next-generation sequencing. (DOCX 92 kb)

## Abbreviations

CT: Computed tomography
DLco: Denotes diffusing capacity of the lung for carbon monoxide
FEV1: Forced expiratory volume in 1 s
FVC: Forced vital capacity
LAM: Lymphangioleiomyomatosis
MRI: Magnetic resonance imaging
mTOR: Mammalian target of rapamycin
RAML: Renal angiomyolipoma
SEGA: Subependymal giant cell astrocytomas
TSC: Tuberous sclerosis complex
