# Supplementary material for: Assessing the outcomes of everolimus on renal angiomyolipoma associated with tuberous sclerosis complex in China: a two years trial
Source: Orphanet J Rare Dis. 2018 Mar 27;13:43. doi: 10.1186/s13023-018-0781-y (PMC5870799; doi:10.1186/s13023-018-0781-y)
Supplement: Supplementary file 1 — Visit Schedule and Assessments. (DOCX 73 kb) [file 13023_2018_781_MOESM1_ESM.docx]

**Supplementary Table 1. Visit Schedule and Assessments.**

|  | **Core period** | | | | **Extended period** | |
| --- | --- | --- | --- | --- | --- | --- |
|  | **Baseline** | **+3m** | **+6m** | **+12m** | **+18m** | **+24m** |
| **Informed consent** | √ |  |  |  |  |  |
| **History/Physical examination** | √ | √ | √ | √ | √ | √ |
| **CBC, blood chemistry, liver and renal functions, blood lipid** | √ | √ | √ | √ | √ | √ |
| **Pulmonary function test** | √ | √^#^ | √^#^ | √^#^ |  | √^#^ |
| **Blood gas analysis at room air** | √ | √^#^ | √^#^ | √^#^ |  | √^#^ |
| **Cranial MRI** | √ | √^#^ | √^#^ | √^#^ |  | √^#^ |
| **Quantitative Chest HRCT** | √ | √^#^ | √^#^ | √^#^ |  | √^#^ |
| **Kidney three-dimensional CT** | √ | √ | √ | √ | √ | √ |
| **Concurrent Medication** | √ | √ | √ | √ | √ | √ |
| **Safety Evaluation** |  | √ | √ | √ | √ | √ |
| **Test drug count and delivery** |  | √ | √ | √ | √ | √ |
| **Telephone follow-up** | Every month^*^ | | | | | |

**^#^**It is not necessary if baseline evaluation does not find abnormal.

**^*^**Content of telephone follow-up includes: (1) drug use and amount remaining, (2) adverse events, (3) disease development, clinic, emergency and hospitalization, (4) concurrent drugs, (5) remind of the next visit.
