# Supplementary material for: Assessing the outcomes of everolimus on renal angiomyolipoma associated with tuberous sclerosis complex in China: a two years trial
Source: Orphanet J Rare Dis. 2018 Mar 27;13:43. doi: 10.1186/s13023-018-0781-y (PMC5870799; doi:10.1186/s13023-018-0781-y)
Supplement: Supplementary file 2 — Physician’s Global Assessment of Clinical Condition. (DOCX 98 kb) [file 13023_2018_781_MOESM2_ESM.docx]

**Supplementary Table 2. Physician’s Global Assessment of Clinical Condition.**

| **Grade** | **Description** | **Response** |
| --- | --- | --- |
| 0 | Completely clear: no evidence of disease (100% improvement) | CCR |
| 1 | Almost clear: very significant clearance (90% to 100%); only traces of disease remains | PR |
| 2 | Marked improvement: significant improvement (75% to 90%); some evidence of disease remains | PR |
| 3 | Moderate improvement: intermediate between slight and marked improvement (50% to 75%) | PR |
| 4 | Slight improvement: some improvement (25% to 50%); however, significant evidence of disease remains | SD |
| 5 | No change: disease has not changed from baseline condition (± 25%) | SD |
| 6 | Worse: disease is worse than at baseline evaluation by 25% or more | PD |

CCR: clinical complete response; PR: partial response; SD: stable disease; and PD: progressive disease.
