# Supplementary material for: Assessing the outcomes of everolimus on renal angiomyolipoma associated with tuberous sclerosis complex in China: a two years trial
Source: Orphanet J Rare Dis. 2018 Mar 27;13:43. doi: 10.1186/s13023-018-0781-y (PMC5870799; doi:10.1186/s13023-018-0781-y)
Supplement: Supplementary file 3 — Mutations detected by next-generation sequencing. (DOCX 92 kb) [file 13023_2018_781_MOESM3_ESM.docx]

**Supplementary Table 3. Mutations detected by next-generation sequencing.**

| **No** | **Sex** | **Age** | **Mutant gene** | **Nucleotide change** | **Protein change** | **Location** | **Mutation Type** |
| --- | --- | --- | --- | --- | --- | --- | --- |
| 1 | Male | 30 | TSC2 | c.4006_4007 insC | p.Ser1336SerfsX78 | EX34/CDS33 | Frameshift |
| 2 | Male | 24 | TSC2 | c.3412C>T | p.Arg1138Ter | EX30/CDS29 | Nonsense |
| 3 | Female | 35 | TSC2 | c.4129C>T | p.Gln1377Ter | EX34/CDS33 | Nonsense |
| 4 | Female | 35 | TSC2 | c.788_789insC | p.Leu263LeufsX75 | EX9/CDS8 | Frameshift |
| 5 | Male | 30 | TSC2 | c.2738_2739 insT | p.Thr913ThrfsX2 | EX24/CDS23 | Frameshift |
| 6 | Female | 24 | TSC2 | c.2319 delA | p.Leu773LeufsX56 | EX21/CDS20 | Frameshift |
| 7 | Female | 23 | TSC2 | EX2_16 DEL | — | EX2_16/CDS1_15 | Deletion |
| 8 | Female | 40 | TSC2 | c.203_204 insA | p.Ala68AlafsX7 | EX3/CDS2 | Frameshift |
| 9 | Male | 24 | TSC2 | c.3750C>G | p.Tyr1250Ter | EX31/CDS30 | Nonsense |
| 10 | Male | 24 | TSC2 | c.4255C>T | p.Gln1419Ter | EX34/ CDS33 | Nonsense |
| 11 | Male | 25 | TSC2 | c.1947-1G>C | — | IN18 | Splice |
| 12 | Female | 20 | TSC2 | c.1507C>T | p.Gln503Ter | EX15/CDS14 | Nonsense |
| 13 | Female | 37 | TSC2 | c.3683_3684 insG | p.Leu1228LeufsX6 | EX31/CDS30 | Frameshift |
| 14 | Female | 34 | TSC2 | c.5024C>T | p.Pro1675Leu | EX39/CDS38 | Missense |
| 15 | Female | 30 | TSC2 | c.3601_3602 insGGCCC | p.Thr1203GlyfsX9 | CDS29 | Frameshift |
| 16 | Female | 28 | TSC2 | c.4926delC | p.Asn1643ThrfsX29 | CDS37 | Frameshift |
| 17 | Female | 46 | TSC2 | c.203_204 insA | p.Ala68AlafsX7 | EX3/CDS2 | Frameshift |
| 18 | Female | 28 | TSC2 | c.5126 C>T | p.Pro1709Leu | EX40/CDS39 | Missense |
